# Characterization and Morphology of Natural Dung Polymer for Potential Industrial Application as Bio-Based Fillers

**DOI:** 10.3390/polym12123030

**Published:** 2020-12-17

**Authors:** Vinayak Fasake, Kavya Dashora

**Affiliations:** Agricultural Nano Biotechnology Lab, Centre for Rural Development and Technology, Indian Institute of Technology, Delhi Hauz Khas, New Delhi 110016, India; Vinayak.fasake@gmail.com

**Keywords:** chemical composition, indigenous cow dung, non-wood pulp, ruminant animal, wheat straw

## Abstract

The modern-day paper industry is highly capital-intensive industries in the core sector. Though there are several uses of paper for currency, packaging, education, information, communication, trade and hygiene, the flip side of this industry is the impact on the forest resources and other ecosystems which leads to increasing pollution in water and air, influencing several local communities. In the present paper, the authors have tried to explore potential and alternate source of industrial pulp through ruminant animal dung, which is widely available as a rural resource in India. Three types of undigested animal dung fibers from Indigenous cow (IDF), Jersey cow (JDF), and Buffalo (BDF) were taken. Wheat straw (WS) was the main diet of all animals. The cellulose, hemicellulose and lignin content for all animal dung samples were found in a range of (29–31.50%), (21–23.50%), and (11–13%), respectively. The abundant holocellulose and low lignin contents are suitable for handmade pulp and paper. Surface characteristics of fodder (WS) and all dung fibers have been investigated using Fourier Transform Infrared Spectroscopy (FTIR), scanning electron microscopy (SEM), and SEM-Energy dispersive X-ray spectroscopy (SEM-EDX). To increase paper production without damaging forest cover, it is essential to explore unconventional natural resources, such as dung fiber, which have the huge potential to produce pulp and paper, reinforcement components, etc.

## 1. Introduction

The pulp and paper industry is one of the biggest industries in the world and a growing portion of the world’s economy. According to the global paper market review 2018, the paper produced and consumed globally was approximately 406 million tons in 2017 [1]. The share of paper and paperboard production in China has increased from 15.3% to 23.5% in five years. This was possible because of the development of the Chinese paper industry due to policymakers, researchers and international producers [2]. India’s share in world production of paper is about 3.7%, with an estimated production of over 20 million tons per annum (2017–2018). Around 50% of this paper is used in major developed countries, with China consuming about 106 million tons. The consumption in the USDA is around 71 million tonnes, and Japan stands third, consuming 27 million tons. Paper consumption in Europe is around 92 million tonnes, whereas the developing and underdeveloped countries, such as Africa, Oceania and Latin America consume about 2% to 8% annually. The high domestic production in developing countries such as China and India cannot satisfy the demand, especially for high-grade paper, due to lack of high-quality raw material and old paper production technology being used in the industry. Therefore, such countries are importing more pulp and paper products than exporting. In the total pulp consumption of the world, the proportions of virgin fiber, recovered fiber and other fiber (non-wood) are 42, 55, and 3%, respectively [3]. Population growth, increasing literacy rate, industrial revolution and booming e-commerce sector in developing countries are some of the key projected reasons for increase in paper and pulp demand per annum globally. With the growing industrial demand, the utilization of wood is increasing and at the same time, non-wood pulp production is also becoming crucial in the countries that do not have enough trees for pulp industry, such as China, India, Pakistan, Egypt, and Columbia. Currently, recycled waste and non-wood biomass with grasses, cereal straws, corn stalks, bamboo, and bagasse, have been found to be suitable alternatives to trees to complete our demand and supply of raw materials in a commercially viable manner. Typically, lignocellulosic biomass contains 30–50% cellulose, 20–35% hemicellulose and 15–30% lignin, which is perfectly matched for various industrial applications [4]. Alternative fibers, such as agricultural biomass resources, have the potential to form pulp for papers thereby substituting forest wood, leading to greater sustainability, industry efficiency and lower climate impacts. Non-timber-based fibers are known to possess a variety of physical and optical properties, which can pave the way for utilizing them as a raw material in the timber dependent pulp and paper industry. Life-Cycle Assessment by tissue manufacturing giant company Kimberly-Clark inferred that, when compared between agricultural residues, softwood pulp, and transportation, the softwood-based pulp consumes the maximum use of fossil fuels for paper production and have the highest greenhouse gas emissions, and agricultural residues consume the least fossil fuels [5].

### Indian Farming, Livestock and Dung Potential

In India, 66.46% of the population reportedly resides in rural areas (World Bank, 2017), where over 15–20% of families are landless and about 83% of the landholders belong to the category of small and marginal farmers [6]. Livestock, being a key source of supplementary income and livelihood, especially for small landholders and the landless rural poor, play an important role in the rural economy of the country [7]. In India, the total livestock the population is approximately 600 million; where the cow and buffalo contribute 35.94% and 20.45% of the total population, respectively, and the supply of raw material (dung) is substantial [8]. In total, 500 million tons (Mt) of gross agricultural residue is generated on an annual basis with wide regional varieties of crop-like cereals, oilseed, pulses, and sugarcane, etc. While a major portion of ruminant livestock in South-East Asia, including India, is based on such cereal crop residues, such as roots, stalks, and leaves [9].

Bovines are typical ruminants with a four-chambered stomach, namely rumen, reticulum, omasum and abomasum [10]. During the digestive process, the raw materials are processed mechanically in the first chamber of rumen followed by the bacterial breakdown of cellulose in the reticulum. The partially digested materials (cud) move back into the mouth and are rechewed and re-swallowed as regurgitated materials to finally break it into further finer forms. Now this enters in the other chambers, namely omasum and abomasum, where most of the moisture from the food is absorbed. In these chambers, digestive enzymes, such as lysozyme and many anaerobic microorganisms, digest the hemicellulose and pectin content of the plant fibers. It can be easily inferred that a high percentage of cellulose is undigested and excreted in cow dung [11,12]. Dung is one of the bioresources of this world which is available on a large scale and is still not fully exploited for its potential. In this way, ruminant animal dung may be considered as an easily available bioresource that holds great potential for sustainable development in the near future [13]. Sustainable conversion of renewable biopolymeric feedstock in environmentally friendly products for diversity and the right applications fit well into the green growth economy [14]. While the policy tends to focus on milk production, dung is already driving an informal economy of national importance, which was largely overlooked. Since 2016, Government of India has also been working on dung collection and its utilization under the scheme of Galvanizing Organic Bio-Agro Resources (GOBAR)-Dhan Yojana. Similarly, Cattle commission named Kamdhenu Aayog is also working on a similar approach to develop a circular economy and strengthen the livelihood through cow dung. The extraction of fibrous material from dung would add to the various uses of cow dung, such as the production of biogas, compost, etc., and keeping the organic matter for their other uses.

In the present paper, the authors have tried to explore the potential of ruminant animal dung as a sustainable and alternate source of non-wood material for the pulp and paper industry. Additionally, the morphology and physical characterization of raw fiber is studied for other applications in the future.

## 2. Material Methods

### 2.1. Sample Collection

One cattle each of three different varieties of Indigenous cow (Hariana breed), Jersey cow (Crossbred *Holstein Friesian*) and Buffalo (Murrah) were selected for an experiment at “Gaushala Mandir”, Gaushala Marg, Kishangar village, New Delhi, Delhi. Wheat straw was the staple food for all the three selected cattle for 5 days. After five days of continuous feeding of the same food material (wheat straw), fresh dung was collected twice a day. Under authors’ observation, the feeding and handling of animals, and collection of dung were done by a trained person from the gaushala.

### 2.2. Preparation of Raw Material

**Figure 1:**
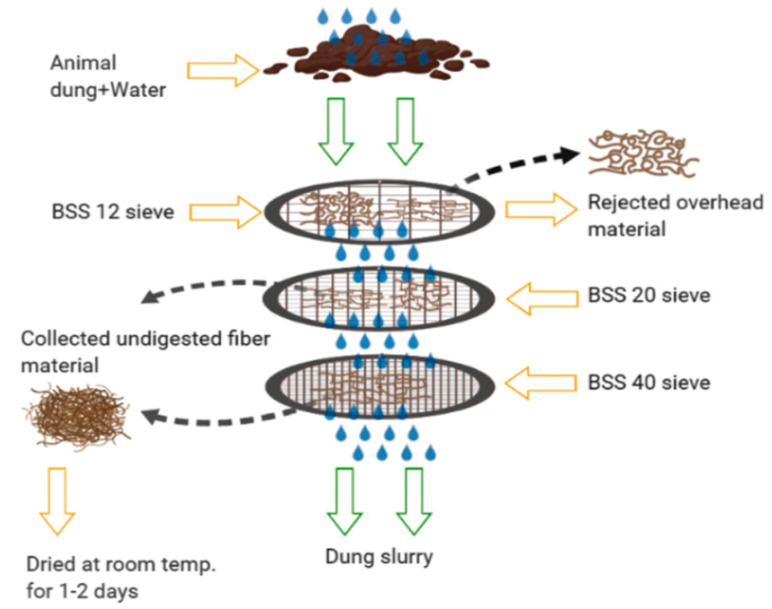
Schematic view of separation of undigested dung material and dung slurry.

**Figure 2:**
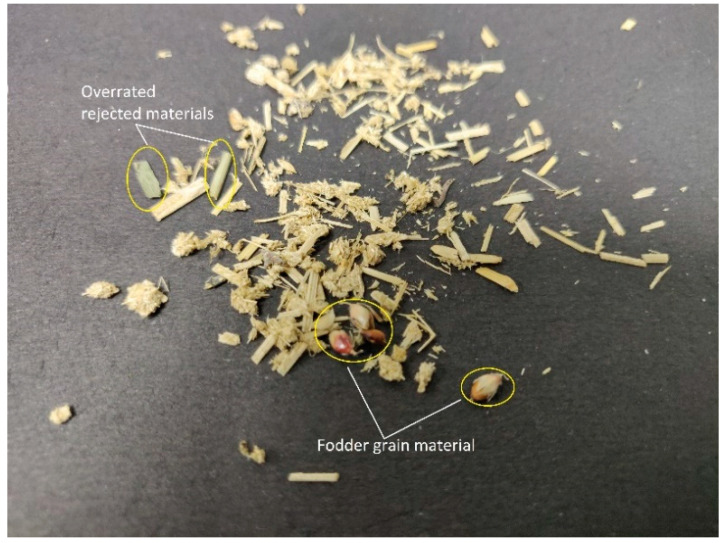
Overrated rejected materials (Green fodder, Grains etc.).

Fresh dung was collected and was passed through standard test sieve BSS 12, BSS 20, and BSS 40, respectively, under the normal flow of showering tap water. A sample obtained after passing through a BSS 12 sieve was rejected because of the presence of large impurities and some fodder straw. Samples obtained after passing from both the BSS 20 and BSS 40 sieves were kept at room temperature for 1 day and overturned occasionally by using sterilized wooden sticks. These samples were then dried at 105 °C for 30 min using hot air oven (Ambay Biotech, Delhi, India) and stored in separate airtight plastic bags under the dry conditions for subsequent experiments. The schematic diagram is shown below in Figure 1; the sample preparation and rejected overrated materials (impurities) are shown in Figure 2.

### 2.3. Proximate and Ultimate Analysis of Raw Dung Fiber

Moisture content and volatile solids were determined by calculating the difference between initial and final value. The total solid contents in raw materials was determined as per the method reported by American Public Health Association [15]. Ash content was determined by (T 211 cm-02) method, reported by TAPPI standards.

### 2.4. Physical Characterization of Raw Animal Dung Fiber

Fiber morphology was analyzed by randomly selecting 100 raw dry fibers. The length of the fiber obtained (maximum, minimum and average) was measured through a Nikon eclipse E200 microscope, Tokyo, Japan. Average maximum and minimum value count were calculated as a maximum and minimum length of fiber and diameter of fiber in millimeter (mm) and micrometer (µm), respectively.

### 2.5. Chemical Characterization

Chemical composition of all the three-animal dung fiber and fodder material (wheat straw) was determined. The extractive substances ware subjected to different liquids according to the some common standards, such as cold-water solubility (Tappi T 207 cm-99), hot-water solubility (Tappi T 207 cm-99), 1% NaOH solubility (Tappi T 212 cm-02), Alcohol–Benzene solubility (Tappi T 204 cm-97). Cellulose, hemicellulose and lignin from the sample were determined using the method published by the National Renewable Energy Laboratory (NREL Protocol, CO, USA, 2012).

### 2.6. Scanning Electron Microscopy (SEM)/Energy Dispersive X-ray Spectroscopy (EDS) Imaging of Dung Fibers

The microstructure of the sample was characterized by scanning electron microscopy (SEM). All the raw fiber material was first converted into a fine powder and then observed on a model TM-3000 scanning electron microscope (HITACHI, Tokyo, Japan) following metal spraying and the fixation of samples on a thin gold coating (Emitech K550X). Similarly, the vertical structure of fiber materials was observed through Carlzeiss EVO18, operated at 20 kV.

### 2.7. Fourier Transform Infrared Spectroscopy (FTIR)

Fourier transform infrared spectroscopy studies on all animal dung fibers and wheat straw. The powdered dried sample placed on a Nicolet iS10 FTIR system (Thermofisher Scientific, Waltham, MA, USA) in the range of 400–4000 wave number.

### 2.8. ICP-MS

The determination of elements in the fibered samples were carried out with inductively coupled plasma ICP-MS Agilent 7900 (Agilent Technologies, Santa Clara, CA, USA before that sample was digested by Microwave reaction system (Anton Paar, Multiwave PRO, Graz, Austria). This method enabled the detection of macroelements (potassium (K), calcium (Ca), magnesium (Mg)), microelements (boron (B), iron (Fe), copper (Cu), manganese (Mn), molybdenum (Mo), zinc (Zn)), and metals/metalloids (aluminium (Al), barium (Ba), bismuth (Bi), cadmium (Cd), cobalt (Co), chromium (Cr), nickel (Ni), lead (Pb), vanadium (V), etc.

### 2.9. Statistical Analyses

The experiments were repeated in triplicate and the results are reported as the mean of replicates with standard deviation (mean ± SD) of the values. The experiments were carried out using completely randomized design with SAS 9.4 software. A level of *p <* 0.05 was considered significant. The data obtained were submitted to analysis of variance, and the least significant differences were used to compare the different treatments individually and in combination both by Duncan methods.

The physical and biochemical characterization of the selected raw material was done to identify the scope of using dung fiber as a potential eco-friendly source for the pulp and paper industry.

## 3. Results and Discussion

### 3.1. Initial Physical Characteristics of Ruminant Animal Waste Dung

#### 3.1.1. Percentage Yield of Raw Material

The retained overrated fibrous material on both the sieves were collected and dried to constant weight. The percentage of semi-digested or broken fodder material (fiber) in different animal dung were obtained below, as shown in Table 1. The small variations were observed due to the different breeds or varieties of animals, diet pattern, animal health and it was mainly depending on water quality, the type of fodder (cellulosic materials) and particle size of fodder. During the experiments, it was observed that buffalo has a slower rumen movement than the cow, which leads to a slower rate of ingesting outflow, takes more time to regurgitate, re-chew and digest [16]. Based on this, it was observed that in the buffalo dung, the size and quantity of undigested fiber material were found to be more in prevalent in buffalo dung as compared to the cow dung. Further, whatever was semi-digested or broken plant material, was excluded as dung and the percentage of such material in different animal dung are shown in Table 1.

#### 3.1.2. Morphological Analysis of All Dung Fibers

**Figure 3:**
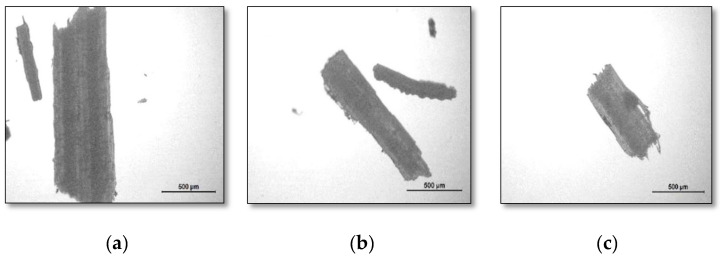
Different dung fiber material exposed upto (500 µm) microscope for easy measurement of average length and diameter. (**a**) Indigenous dung fiber (IDF), (**b**) Jersey cow dung fiber (JDF), (**c**) buffalo dung fiber (BDF).

A total of 100 randomly chosen fibers were scrutinized, as shown in Figure 3. The raw fiber material particle size in terms of length (mm) and diameter (µm) are shown in Table 2. The fiber length and diameter were slightly increased in JDF than IDF and also increased in BDF compared to JDF. In ruminant animals, the reduction in the size of the food materials depends on fodder type, size of fodder crop, cud-chewing time, health and comfortable herd of the animal, etc. There was no major difference between IDF, JDF, and BDF samples, but the raw IDF and JDF fiber materials had a softer texture as compared to BDF.

#### 3.1.3. Scanning Electron Microscope (SEM)

**Figure 4:**
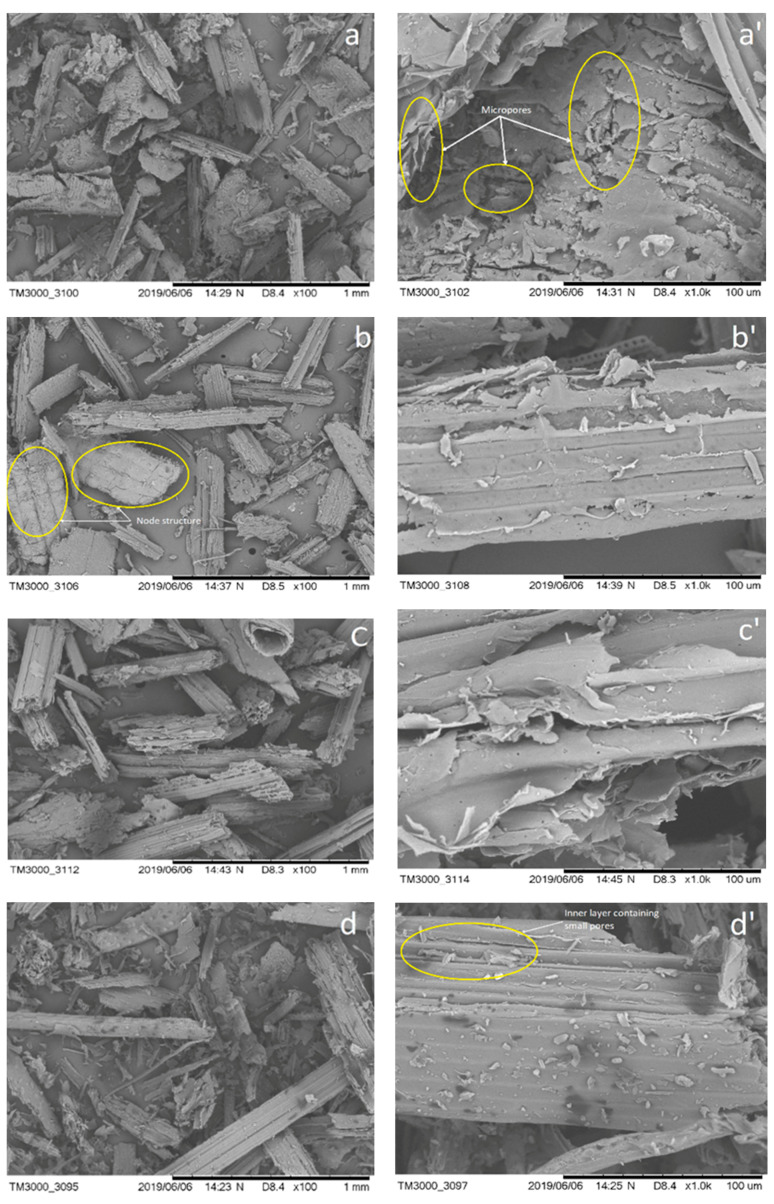
Morphologies of the Indigenous cow dung fiber (**a**,**a’**), Jersey cow dung fiber (**b**,**b’**), Buffalo dung fiber (**c**,**c’**) and wheat straw (**d**,**d’**) Left side: ×100, Right side: ×1000.

Scanning electron microscopy (SEM) was used to analyze raw dung fiber where wheat straw (WS) used as a fodder material to all three animals at magnifications of 100×, and 1000×, respectively, as shown in Figure 4, after being digested in their rumen and excreted in the form of dung. The above samples were not subjected to any physical or chemical processes. In Figure 4 the rough structure and uneven surface morphology included small holes and trenches in all above samples were observed. The skeleton structures were observed due to the presence of cellulose content. Cattle dung fiber was slightly exposed on their outer layers and some node-like structures and micropores were seen but there was no significant difference seen between wheat straw and dung fibers which could have increased the internal adhesive bonding between the different fiber to fiber interfaces and absorb some water content during the papermaking process [17,18]. Figure 4c,d shows that the inner layers are composed of interconnected thin-walled tubules [19] In Figure 4, there are some small black spots observed. Some of the literature showed it as lignin content, pectin and some oily or waxy residues [20]. Since it is not biochemically tested, the possibility of impurities and sediments, such as soil dust, clay particles, other undigested grains, etc. attached to the wheat straw cannot be ruled out.

**Figure 5:**
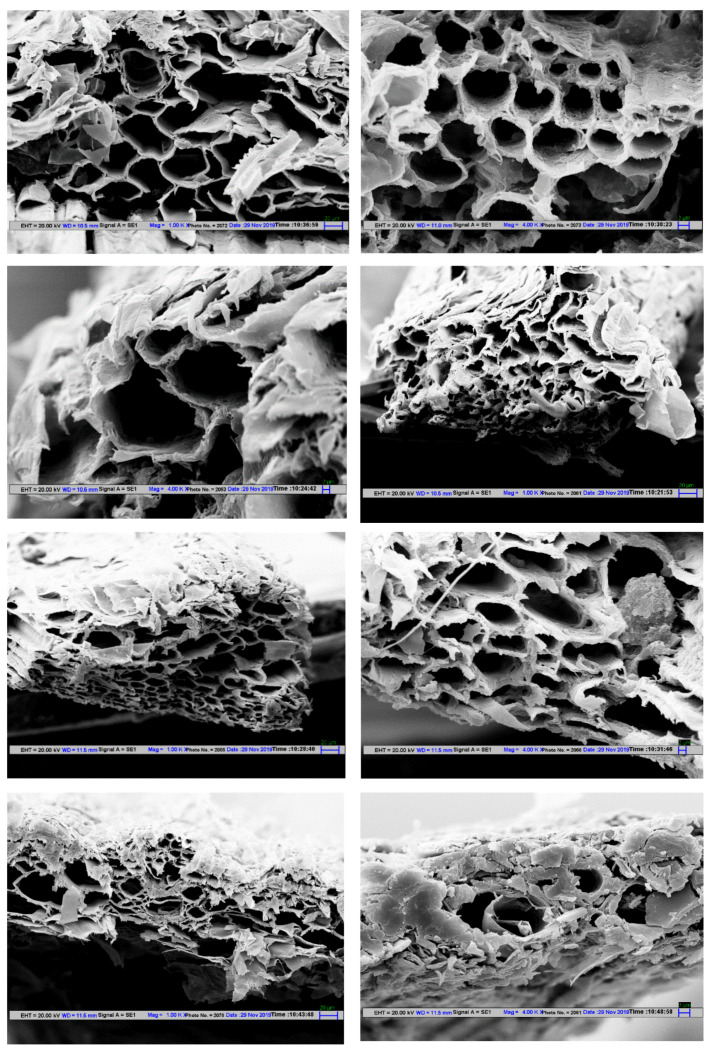
Scanning Electron Microscopy (SEM) images of a vertical cross section of Indigenous cow dung fiber (IDF), Buffalo dung fiber (BDF), Jersey cow dung fiber (JDF) and wheat straw (WS) (untreated) (Left side: ×1000, Right side: ×4000).

Scanning electron microscopy (SEM) analysis was performed using a Carlzeiss EVO18, operated at 20 kV. The fiber samples were placed vertically over the silver tape and coated gold with a sputter coater (Emitech K550X). These secondary SEM images were taken at up to ×4000 magnification to examine the structural morphology, as shown in Figure 5. Evidently, each sample exhibited a porous structure including pore size, shape, and distribution. Comparing WS with all dung fibers samples, it is clear that IDF, JDF and BDF contain larger pore size with broad openings, while the WS had the presence of smaller pores that were compact in nature.

#### 3.1.4. Energy-Dispersive Spectrometer (EDX)

**Figure 6:**
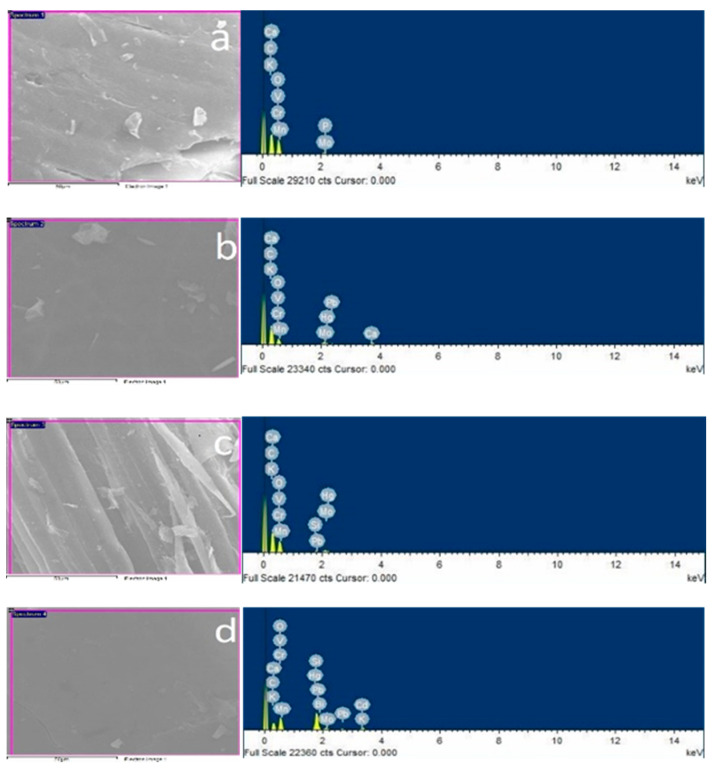
Scanning Electron Microscopy (SEM)/Energy Dispersive X-Ray (EDX) spectrum of all dung fibers and wheat straw: (**a**) IDF, (**b**) JDF, (**c**) BDF and (**d**) WS.

The cattle dung fiber and WS were examined under the SEM micrograph and corresponding EDX spectrum (Quanta 200 F) for determination of non-metals and any other solid biomass present on the surface of all samples. Figure 6 shows Ca, K, V, Mn, Cr, Pb, P, Mo, Bi, Hg, Si. After the confirmation of the presence of these elements, all samples were examined under inductively coupled plasma mass spectrometry (ICP-MS) and the results are shown in Table 6.

#### 3.1.5. Fourier-Transform Infrared Spectroscopy (FTIR)

**Figure 7:**
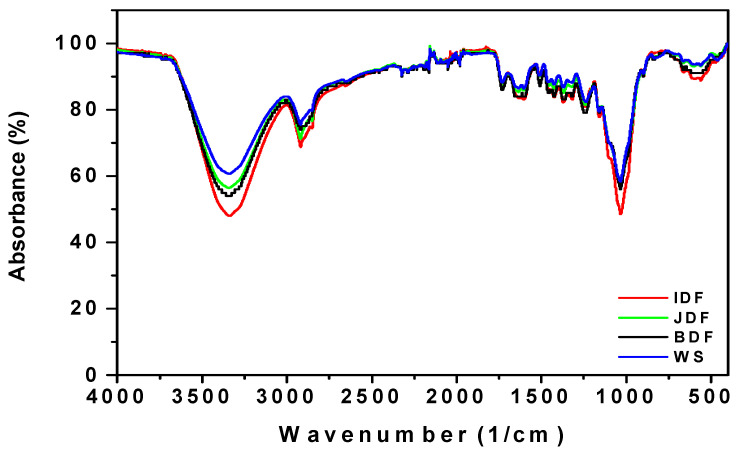
Fourier transform infrared spectroscopy (FTIR) spectra of IDF, JDF, BDF and WS.

Fourier-Transform Infrared Spectroscopy (FTIR) was used to identify the functional group present in a cattle dung material. The FITR spectrum of IDF, JDF, BDF, and WS showed similar patterns with notable bands, as exceptionally presented in Figure 7. The main absorbance bands were considered. The spectrum displayed large band adsorption peaks at 3430 cm^−1^ which represent OH stretching vibration due to hydrogen vibrations of the OH groups of alcohols, phenols or carboxylic acid. The peak at 2920–2870 cm^−1^ is due to the characteristics of –CH vibrations of aliphatic groups. The band centered between 2360–2340 cm^−1^ gave C=C stretching of terminal alkynes. Subsequently, most of the organic compounds that encompass one or more heteroatoms, such as oxygen, nitrogen or sulphur exhibit corrosion inhibition ability in acidic media. Organic molecules that have groups, such as –OH, −CHO, –COOH, –CN, –SCN, –CO, –NH_2_, –SO_3_, double or triple bonds or unpaired electrons, have been attested to interact easily with metals, thereby leading to the protection of metals in aggressive medium.

### 3.2. Proximate and Ultimate Analysis

The proximate and ultimate analysis is commonly used to characterize solid biomass samples for determining the behavior and the elemental composition of biomass materials when it is heated. Table 3 show the proximate and ultimate analysis of raw animal dung fiber materials and wheat straw. The volatile contents of all dung fiber and WS were determined on a dry basis. The lowest volatile content was found in the IDF—i.e., 92.85 ± 0.7%.—and the highest volatile content in the BDF—i.e., 95.91 ± 0.57%.

The results in Table 3 show variation from the existing studies in the scientific literature due to different or uneven microbes and enzymatic degradation of the fodder crop inside the rumen of animal. Comparing the existing and present study, it can be concluded that when any fodder crop is passed through the ruminant animal system then the percentage of carbon and hydrogen content is slightly deflected. The moisture content in all animal dung fibers was observed to be very less as compared to the wheat straw. Some studies reported that, due to low moisture content, such material is also suitable for the pyrolysis processes [21]

### 3.3. Chemical Characterization

#### 3.3.1. Solubility

The soluble extracts in ethanol-benzene show the lower level of extractable content in all dung fibers varied from 1.35–3%, such as low molecular weight carbohydrates, salts, waxes, fats, and resinous substances. Generally, the ethanol-benzene solubility consists of inorganic compounds, tannins, gums, waxes, fats, sugars, coloring matter, starch, salts, some low molecular carbohydrates and protein which could affect the pulping process as a whole [22,23,24]. The alcohol-benzene, soluble in IDF and JDF, were almost the same but were observed to be at a higher level in WS, and on a lower level in BDF. The higher content of the solvent extracts in the pulp can have an opposite impact on the functioning of the process equipment, resulting in a reduction in water absorbency [25]. These values were within limits of many other materials, such as Brutia pine 1.94 [26], Bamboo 2.3% [27], Giant reed 4.55–6.34% and Napier grass 3.1% [28].

The high solubility in 1% sodium hydroxide indicated the extent of fiber degradation during alkaline pulping (kraft and soda process) resulting in the screened yield of chemical pulp lesser than expected. In our samples, BDF indicates lower solubility, while IDF and JDF had nearly followed each other. The 1% NaOH solubility in our all samples are similar to the other studies, such as those of [29,30] Schott (2000) and Deniz et al. (2004), who found 41–42.8% and 40.59%, respectively. The NaOH solubility is within the range of values identified by most non-wood materials for example lemongrass 30.64% [31] (Kaur and Dutt, 2013), Cotton stalks 39.60%, some wood material, such as olive wood 30% [32] (Jimenez et al. 2008).

Table 4 show the percentage of hot water solubility in all three dung samples and WS ranged from 12–20.5%. The wheat straw was observed to have slightly more solubility, which indicated that can easily convert cattle dung fibers into the pulp as compared to WS. The high hot water solubility could be consuming more pulping reagents due to the higher content of sugars, coloring matters, starch and proteins resulting in content low pulp yield after the pulping process. The cold water extractives also vary from 6–8%. Both cold and hot extractives did not vary much between animal dung fibers and WS were in ranges of value already identified with other non-wood materials but higher than those of wood. Therefore, from the chemical composition analysis, the pulp yield of animal dung fiber is expected to be the same as other non-wood materials, including agricultural residues.

#### 3.3.2. Major Chemical Composition Cellulose, Hemicellulose and Lignin Content

The amounts of holocellulose and lignin content in the selected raw materials are the most important indicators of the use of raw materials in paper production. The cellulose content of different dung fibers and wheat straw revealed that wheat straw showed the highest value at 39.36 ± 0.64, whilst IDF, JDF, and BDF were almost near to each other. Cattle chew the lignocellulosic material and during the digestion process, this food is digested through some acids, digestive juices, microbes and enzymes in their stomach. The results also showed that the cellulose content was slightly deflected in all three animal dung samples as compared to fodder crop (WS), indicating that a lot of cellulosic material was retained into dung in the forms of small fibers, as shown in Table 5. It was also indicated that nearly 20% of the cellulose material was dissolved inside the rumen of the animal and it was absorbed by the body.

Mean values for hemicelluloses content have been shown in Table 5. The highest hemicelluloses content was observed in WS (23.65 ± 1.02) followed by IDF (23.46 ± 0.97%) and JDF (22.07± 0.56%) whilst the lowest was found in BDF (21.03 ± 0.34%). Some of the studies showed that hemicellulose content for wheat straw was 18.33% [33], whereas [34] reported that hemicellulose content for wheat straw was 28.95%. It has been shown that the more hemicelluloses in the pulp are responsible for its swelling behavior, which is important for the vital mechanical strength properties, including tensile, burst indexes, and double folds.

Mean values regarding lignin content in different dung fiber material and wheat straw have been presented in Table 5. WS shows maximum lignin content—i.e., 13.91 ± 0.52%—while BDF was at the bottom with 11.97 ± 0.71% and JDF and IDF were found to be very close to each other. The results of the present study are very close to the studies reported by [35,36]. They reported that lignin content in wheat straw was 15–20%, whereas some authors explicated that lignin content in wheat straw was 18.9% [37]. The cellulose and hemicellulose contents in all samples were approximately close to those of wood. [38] Some studies reported 42.68% cellulose and 24.82% hemicellulose in spruce wood. Our results also indicated the low lignin content in our experimental samples as compared to wood, which is a characteristic feature of agro-residues.

In the case of ash, the highest content (7.15 ± 0.77%) was shown by WS, followed by IDF (7.06 ± 0.77%) and JDF (5.49 ± 0.08%), while the lowest content was shown by BDF (4.09 ± 0.57%). The cattle dung fiber in the study reported lower ash content than other non-wood fodder materials, such as corn stover 7.82%, Bagasse (8.02%), Rice straw (20.02%), etc. Lower ash content indicates maximum pulp yield with good quality of paper. Conversely, higher content of ash causes severe scaling problems during pulping, high fouling and corrosion tendencies and subsequent refining and recovery of cooking liquor [39]. (The chemical composition of cow dung may vary and may depend on the source from which the cow dung is obtained. By keeping in mind the results, cellulose, hemicellulose, lignin and ash content present in wheat straw and different animal dung fibers are suitable for pulp preparation, owing to their high holocellulose and low ash and lignin content. This feature can be considered as a serious advantage when looking for new alternative sources of fibers for papermaking.

#### 3.3.3. ICP-MS

The ICP-MS technique was used for the element analysis or chemical characterization of the biomass sample. High concentration of Mg was characteristic of all dung fiber sample and wheat straw. Macro-elements in all analyzed straws followed the sequence Ca < K < Mg. The heavy metals, such as Pb, have a significant toxic effect. The heavy metals chiefly include Pb, Ni, Cd, Cr, Al, Co, Ba, and V. The heavy metals, viz., Cd, Pb, are considered most toxic to humans, animals, fishes, and the environment. The element content found in dung slurry was found to be more than the dung fiber (Table 6).

## 4. Conclusions

Pulp production depends heavily on large tons of timber sources, which are done by razing natural forests. In some other areas, to meet the industrial demand, a large number of pulping trees are planted, which is again a non-native habitat to the world’s most critically endangered flora and fauna. This deforestation mostly happens in the biodiversity hotspots, and rain forest areas losing their land at an alarming pace of approximately 8 million hectares per year. Lignocellulosic agro-residues are the abundant, non-timber based and the cheapest source of organic raw materials. The Indian subcontinent is blessed with large cattle population and rearing cattle had been an integral part of our civilization. The cattle dung fiber is potentially an effective source of an alternative non-wood cellulosic material for handmade pulp and paper production. From the present study, the chemical composition indicated an appropriate cellulose and hemicellulose content similarly, low lignin and ash content, which is considered as a vital industrial criterion for the production of high-quality pulp and paper. Scanning electron microscopy (SEM) analysis shows the layer matrix, skeleton structure and honey-comb-like packed arrangement of fiber making. It is a very suitable raw material for pulp and as a blending material with virgin pulp, recycled pulp, cotton rags, and any other higher cellulosic materials in the papermaking process, as per the customer demand. It is also clear that the natural dung polymer should be used as a biobased filler with any other higher cellulosic blending materials. The authors also concluded that, instead of direct conversion of fodder biomass into pulp and paper, if the digested lignocellulosic material from an animal is used, the non-milking and old animal will also find some industrial use and the cutting of trees for paper can be avoided. Presently, the Indian paper industry is in a nascent stage and is still experimenting with potentially better ways to develop a sustainable industry which is eco-friendly and also resource friendly. This will also help to develop circular economies for the farmers or cattle owners having dry or non-milking cattle in their herds.

## Figures and Tables

**Table 1:** Percent of undigested raw material from fresh dung.

Sample Name	% of Fiber (db)
IDF	10.08 ± 0.06 ^b^
JDF	11 ± 0.4 ^a^
BDF	11.5 ± 0.08 ^a^

Indigenous cow dung fiber (IDF); Jersey cow dung fiber (JDF); Buffalo dung fiber (BDF); Dry basis (db). ±Standard deviation from the mean; the values with same superscripts were found to be statistically alike at *p* ≤ 0.05 in the same column and variety followed by different alphabets differ significantly. a > b.

**Table 2:** Measurement of raw fibers.

Sample Name	Min. Length of Fiber (mm)	Max. Length of Fiber (mm)	Avg. Length of Fiber (mm)	Min. Diameter of Fiber (µm)	Max. Diameter of Fiber (µm)	Avg. Diameter of Fiber (µm)
IDF	0.09 ± 0.03 ^a^	1.6 ± 0.09 ^b^	0.85	51 ± 1.71 ^c^	1803 ± 1.12 ^c^	927
JDF	0.11 ± 0.02 ^a^	2.1 ± 0.18 ^a^	1.11	57 ± 1.26 ^b^	1833 ± 1.71 ^b^	945
BDF	0.12 ± 0.04 ^a^	2.26 ± 0.06 ^a^	1.20	68 ± 1.61 ^a^	1892 ± 1.18 ^a^	980

Indigenous cow dung fiber (IDF); Jersey cow dung fiber (JDF); Buffalo dung fiber (BDF); the values with same superscripts were found to be statistically alike at *p* ≤ 0.05 in the same column and variety followed by different alphabets differ significantly. a > b > c.

**Table 3:** Proximate and ultimate properties of basic raw material (% wt. as dry).

Sr.N	Parameters (on Dry Weight Basis of Samples)	IDF	JDF	BDF	WS
Proximate properties					
1.	Moisture (%)	5.40 ± 1.22 ^b^	6.14 ± 0.76 ^ab^	6.35 ± 0.49 ^a^	6.47 ± 1.33 ^a^
2.	Total solids (TS) (%)	94.60 ± 1 ^a^	93.86 ± 0.62 ^ab^	93.65 ± 0.40 ^b^	93.53 ± 1.09 ^b^
3.	* Volatile solids (VS) (%)	92.85 ± 0.7 ^c^	94.51 ± 0.08 ^b^	95.91 ± 0.57 ^a^	92.94 ± 0.77 ^c^
Ultimate properties					
4.	** C (%)	44.54 ± 0.90 ^a^	45 ± 0.75 ^a^	45.02 ± 0.70 ^a^	45.82 ± 0.71 ^a^
5.	** H (%)	5.82 ± 0.65 ^a^	5.92 ± 0.79 ^a^	5.89 ± 0.50 ^a^	6.69 ± 0.40 ^a^
6.	** N (%)	0.48 ± 0.09 ^a^	0.46 ± 0.10 ^a^	0.50 ± 0.08 ^a^	0.51 ± 0.07 ^a^

Indigenous cow dung fiber (IDF); Jersey cow dung fiber (JDF); Buffalo dung fiber (BDF) and wheat straw (WS). ±Standard deviation from the mean. The values with same superscripts were found to be statistically alike at *p* ≤ 0.05 in the same row and variety followed by different alphabets differ significantly. a > b > c. (VS) was considered on the basis of total solids percentage, * Elemental mol %.

**Table 4:** Chemical composition of animal dung fiber and WS.

Sample Name (Percentage Based on Bone Dry Weight)	Ethanol-Benzene Solubility, % (1:1 *v/v*)	1% NaOH Solubility, %	Hot Water Solubility, %	Cold Water Solubility, %
IDF	1.64 ± 0.08 ^c^	42.20 ± 0.93 ^a^	13.5 ± 0.15 ^c^	6 ± 0.08 ^c^
JDF	1.35 ± 0.07 ^c^	41.56 ± 0.74 ^ab^	13 ± 0.16 ^d^	7 ± 0.15 ^b^
BDF	2 ± 0.12 ^b^	40.02 ± 0.33 ^b^	14 ± 0.12 ^b^	8 ± 0.12 ^a^
WS	3.07 ± 0.24 ^a^	40.10 ± 0.28 ^b^	20 ± 0.37 ^a^	7 ± 0.16 ^b^

Indigenous cow dung fiber (IDF); Jersey cow dung fiber (JDF); Buffalo dung fiber (BDF) and wheat straw (WS). ±Standard deviation from the mean. The values with same superscripts were found to be statistically alike at *p* ≤ 0.05 in the same column and variety followed by different alphabets differ significantly. a > b > c > d.

**Table 5:** Lignocellulosic content of different animal dung fibers and wheat straw.

Sample	Cellulose (%)	Hemicellulose (%)	Lignin (%) *	Ash (%)
IDF	31.34 ± 0.91 ^b^	23.46 ± 0.97 ^a^	12.96 ± 0.69 ^ab^	7.06 ± 0.7 ^a^
JDF	29.94 ± 1.65 ^b^	22.07 ± 0.56 ^ab^	12.06 ± 0.74 ^b^	5.49 ± 0.08 ^b^
BDF	31.19 ± 0.82 ^b^	21.03 ± 0.34 ^b^	11.97 ± 0.71 ^b^	4.09 ± 0.57 ^c^
WS	39.36 ± 0.64 ^a^	23.65 ± 1.02 ^a^	13.91 ± 0.52 ^a^	7.15 ± 0.77 ^a^

Indigenous cow dung fiber (IDF); Jersey cow dung fiber (JDF); Buffalo dung fiber (BDF) and wheat straw (WS). ±Standard deviation from the mean. The values with same superscripts were found to be statistically alike at *p* ≤ 0.05 in the same column and variety followed by different alphabets differed significantly. * Lignin (%) = acid soluble lignin (%) + acid insoluble lignin (%). a > b > c.

**Table 6:** Nutrient content present in Wheat straw, Indigenous cow dung fiber (IDF), Jersey cow dung fiber (JDF), and Buffalo dung fiber (BDF).

Element	IDF	JDF	BDF	WS
	µg/gm			
**Macronutrient**				
Mg	1694.9	2358.3	1786.4	2423.7
K	27.4	29.7	35.2	2189.3
Ca	64.4	65.1	71.2	75.0
**Micronutrient**				
Fe	220.7	151.6	479.5	652.9
Zn	23.3	23.6	23.6	30.8
Mn	11.5	11.7	8.5	11.6
B	223.1	196.3	222.1	212.6
Mo	0.8	0.6	0.8	1.8
Cu	4.2	5.7	7.2	16.6
**Heavy metal**				
Pb	4.7	9.3	8.7	9.6
Ni	0.4	0.3	2.4	2.2
Cd	0.2	0.1	0.0	0.2
Cr	0.7	0.5	4.5	4.2
Al	38.7	39.1	56.9	74.2
Bi	0.0	0.0	0.0	0.0
Co	0.1	0.1	0.1	0.2
Ba	10.7	14.6	23.0	44.6
V	0.3	0.3	0.6	0.5

Indigenous cow dung fiber (IDF); Jersey cow dung fiber (JDF); Buffalo dung fiber (BDF) and wheat straw (WS).
